# A New Structure-Activity Relationship (SAR) Model for Predicting Drug-Induced Liver Injury, Based on Statistical and Expert-Based Structural Alerts

**DOI:** 10.3389/fphar.2016.00442

**Published:** 2016-11-22

**Authors:** Fabiola Pizzo, Anna Lombardo, Alberto Manganaro, Emilio Benfenati

**Affiliations:** Department of Environmental Health Sciences, IRCCS – Istituto di Ricerche Farmacologiche “Mario Negri”Milan, Italy

**Keywords:** hepatotoxicity, structural alerts, chemical clustering, structure-activity relationship, drugs

## Abstract

The prompt identification of chemical molecules with potential effects on liver may help in drug discovery and in raising the levels of protection for human health. Besides *in vitro* approaches, computational methods in toxicology are drawing attention. We built a structure-activity relationship (SAR) model for evaluating hepatotoxicity. After compiling a data set of 950 compounds using data from the literature, we randomly split it into training (80%) and test sets (20%). We also compiled an external validation set (101 compounds) for evaluating the performance of the model. To extract structural alerts (SAs) related to hepatotoxicity and non-hepatotoxicity we used SARpy, a statistical application that automatically identifies and extracts chemical fragments related to a specific activity. We also applied the chemical grouping approach for manually identifying other SAs. We calculated accuracy, specificity, sensitivity and Matthews correlation coefficient (MCC) on the training, test and external validation sets. Considering the complexity of the endpoint, the model performed well. In the training, test and external validation sets the accuracy was respectively 81, 63, and 68%, specificity 89, 33, and 33%, sensitivity 93, 88, and 80% and MCC 0.63, 0.27, and 0.13. Since it is preferable to overestimate hepatotoxicity rather than not to recognize unsafe compounds, the model's architecture followed a conservative approach. As it was built using human data, it might be applied without any need for extrapolation from other species. This model will be freely available in the VEGA platform.

## Introduction

Drug-induced liver injury (DILI) are detrimental adverse effects caused by marketed drugs toward patients' liver (Przybylak and Cronin, 2012). DILI is a major challenge to the pharmaceutical industry, regulatory bodies and physicians (Chen et al., 2014a). Despite pre-clinical and clinical safety assessment of drug candidates, DILI is often the reason for drug failure and consequently for post-approval withdrawal from the market (Egan et al., 2004). The *in vivo* studies during the drug development process are probably able to detect only half of all the human hepatotoxic compounds and *in vitro* studies correctly identify no more than 60% (Ozer et al., 2008; Blomme et al., 2009; Laverty et al., 2010). Besides the economic costs, the late discovery of hepatotoxicity of drugs may have serious health consequences for humans (Howell et al., 2012). DILI is a matter of concern since it is the main cause of acute liver injury (Vinken, 2015). It was calculated that DILI was responsible for half the cases of acute liver failure in the United States (Holt and Ju, 2005).

The liver is the most important organ involved in drug toxicity since functionally it lies between the site of absorption and the systemic circulation (Russmann et al., 2009). This unique position in the body means it receives blood from the gastrointestinal tract and the abdominal space before it is pumped into the general circulation. Thus, when a drug enters the body orally, it is totally or partially absorbed in the gastrointestinal tract and then reaches the liver. In addition, when a drug reaches the general circulation, it is extracted and metabolized by the liver (Roberts et al., 2010), which is the main site for the metabolic activity and elimination of xenobiotics (Russmann et al., 2009). Generally, metabolic transformation leads to the formation of molecules that are no longer—or are less—biologically active, so they are excreted more easily from the body; however, in some cases the metabolic activity of the liver produces substances that are more toxic and reactive than their parent compound (Williams and Park, 2003).

DILI is commonly classified as intrinsic or idiosyncratic. In the first case the hepatotoxicity is caused by the parent compound and/or indirectly by its metabolites. This toxicity is generally dose-dependent and can often be foreseen. Idiosyncratic hepatotoxicity, however, is related to an abnormal reaction to a drug that is not dose-dependent. It generally damages only a limited numbers of people who are hyper-sensitive to a substance, with no specific connection to its pharmacological toxicity. Individual variability and susceptibility to injury make it hard to predict (Cheng and Dixon, 2003; Russmann et al., 2009).

Hepatotoxicity may occur in several ways depending on the different mechanisms of action. For example liver steatosis is caused by abnormal synthesis and elimination of lipids that accumulate in the liver cells interfering with the normal cell activity (Tolman and Dalpiaz, 2007); cholestasis reflects the accumulation of bile acids in the hepatocytes (Padda et al., 2011); liver fibrosis is the excessive accumulation of extracellular matrix proteins including collagen (Bataller and Brenner, 2005).

The hepatic transaminase levels offer a valuable indicator of liver injury. Alanine and aspartate aminotransferase (ALT and AST), alkaline phosphatase (ALP), total bilirubin (TBIL) and γ-glutamyltransferase (GGT) are considered the reference biomarkers and are widely employed for the detection of DILI, providing supporting information in pre-clinical and clinical toxicity studies for drug development (US FDA, 2009; Tonomura et al., 2015). However, they are not always specific and sensitive in recognizing liver diseases provoked by DILI or other causes such as viruses (Przybylak and Cronin, 2012). Gene-expression profiling has now been proposed for more accurate evaluation of DILI (Blomme et al., 2009).

The absence of well-defined specific diagnostic biomarkers for the evaluation of hepatotoxicity (Padda et al., 2011) helps explaining the limited availability of homogeneous data needed for modeling (Cronin and Schultz, 2003). Furthermore, DILI is affected by individual factors such as sex, age, race, health, genetic polymorphism and environment (Pirmohamed, 2006; Greene et al., 2010) which make the few data uncertain. DILI is therefore poorly understood and hard to predict. Early identification of DILI is essential in order primarily to increase drug safety but also to reduce the costs of drug development. Besides *in vitro* techniques which anyway are expensive and time-consuming, interest is rising in computational tools for predicting toxicity that can evaluate and screen large numbers of compounds in a limited time and affect the attrition rates of compounds in drug discovery and development phases (Muster et al., 2008; Valerio, 2009).

Commercial software exists for the prediction of human toxic endpoints such as mutagenicity, carcinogenicity, developmental and reproductive toxicity, skin and eye irritation. However, the prediction of toxicity at organ level is still a challenge on account of the complex intrinsic nature of mechanisms of toxicity and the paucity of reliable *in vivo* and *in vitro* data (Cheng and Dixon, 2003). Despite the objective hurdles to modeling DILI, some *in silico* tools for the prediction of hepatotoxicity have been developed through most of them are commercial. The models for *in silico* assessment of hepatotoxicity have been recently reviewed by Przybylak and Cronin (2012) and Chen et al. (2014b).

Among computational models, quantitative structure-activity relationship (QSAR) and structure-activity relationship (SAR) are the most used ones. QSAR models quantitatively examine the toxicological activity of a compound starting from its chemical structure, on the principle that similar chemical substances should have similar biological behavior. SAR focuses on the rule determining the relationship, as a classifier (Pery et al., 2009; Lombardo et al., 2014). Considering the model structure, *in silico* models can be divided in two main groups: statistical and expert-based. In the first case the models are built on the basis of an automated algorithm; in the second case the human expert, exploiting his/her understanding of toxicological mechanisms, outlines the relationship between the chemical structure and the biological activity (Przybylak and Cronin, 2012). The commercial software Derek for Windows (Lhasa Limited) and CASE Ultra (MultiCASE Inc) contain modules for the prediction of hepatotoxicity. Derek for Windows is based on sub-structure related to a toxicological activity (structural alerts, SAs) and CASE Ultra is a statistical model. Besides software, other *in silico* models based on SAs have been recently described in the literature (Egan et al., 2004; Marchant et al., 2009; Greene et al., 2010; Hewitt et al., 2013).

Here we describe a new SAR model for the prediction of hepatotoxicity based on DILI human data. This model was built by developing automatically and manually-extracted SAs, which are chemical sub-structures linked to a particular activity or toxicity. The use of human data for building the model means the information provided can be used without the need to extrapolate the results from different species, reducing the uncertainty linked to inter-species variability. Furthermore, this *in silico* model can be used as alternative to animal testing for screening purposes and will be implemented in the VEGA platform (http://www.vega-qsar.eu/) and will be freely available to users.

## Materials and methods

### Hepatotoxicity data collection

The first step was to collect data for modeling. Few public datasets on DILI are available. We focused on the following data sources since they were easily detectable and downloadable from the web and they were reliable since already used by other authors (Chen et al., 2013; Hewitt et al., 2013; Zhang et al., 2016).

The first was Fourches et al. (2010), which contains 950 hepatotoxicity data (drugs) on humans, rodents and non-rodent species. These were extracted through a data mining approach based on a combination of lexical and linguistic methods and ontological rules in order to link substances to a series of liver diseases, searching the open literature. This database contains data from *in vitro* and *in vivo* studies and follows a simple classification approach: if DILI effects are reported for a compound it is labeled as toxic, otherwise as non-toxic. More details can be found in Fourches et al. (2010). We selected only data referring to humans (650 data) and eliminated the rest.

The second source was the United States Food and Drug Administration (US FDA) Human Liver Adverse Effects Database. This contains 631 unique pharmaceuticals, 491 of which (non-proprietary data) have adverse drug reaction data for one or more of the 47 liver effects Coding Symbols for Thesaurus of Adverse Reaction (COSTAR) term endpoints (Matthews et al., 2004). For each compound there is an overall activity category (A for active, I for inactive and M for marginally active) referring to five hepatic endpoints: ALP, AST, ALT, lactate dehydrogenase (LDH) and GGT increase. Since only two compounds were labeled as M we eliminated them in order to reduce the uncertainty of the data set.

We merged the two data sets comparing the chemical structures of the compounds by using the software described in Floris et al. (2014). This tool uses multiple combinations of binary fingerprints and similarity metrics for computing the chemical similarity between compounds. In our combined dataset (950 compounds from (Fourches et al., 2010) and 491 from US FDA), we identified 191 duplicated compounds (16.7%). Among these we eliminated and excluded from further analysis those compounds with contrasting experimental values (100 chemicals, 52.4%) and we considered once those chemical with concordant experimental activity (91 compounds, 47.6%, 59 labeled as hepatotoxic, 65%). After concordance analysis we obtained a unique list of 950 compounds. The final data set was fairly balanced, with 510 compounds labeled as hepatotoxic and 440 non-hepatotoxic. We randomly split the data set into training (760 compounds, 80%) and test sets (190 compounds, 20%).

To compile the external validation set, we used the Liver Toxicity Knowledge Base (LTKB) Benchmark Dataset developed by the US FDA. This dataset contains 137 drugs labeled as most-DILI-concern since severe adverse effects are reported for them; 85 less-DILI-concern drugs whose DILI events are mild and 65 compounds labeled as no-DILI-concern since they do not contain any DILI indication (Chen et al., 2011). We considered only those compounds labeled as most-DILI-concern (hepatotoxic) or no-DILI-concern (non-hepatotoxic). We eliminated those compounds already present in the training or test set and we finally obtained a dataset of 101 chemicals, 69 of which were labeled as hepatotoxic and 32 as non-hepatotoxic that we used for testing the performance of the model.

The complete list of compounds used in this work is provided in the supporting information (Data Sheet 1).

### Manual extraction of SAs

#### Unsupervised chemical similarity-based clustering

To identify SAs for hepatotoxicity we created clusters of substances sharing similar chemical structure. This enabled us to hypothesize the presence of toxicity based on common structural features and to group all compounds with the same scaffold but different substituent groups.

We used the similarity index (SI) developed within the VEGA platform (http://www.vega-qsar.eu/). This SI, described in Floris et al. (2014), provides a quantitative measurement ranging from zero to one (where one means that compounds have the same structure) and takes into account all structural features of a molecule. For its calculation, a fingerprint and three molecular descriptors based on structural keys are combined with different weights of importance. Thus, this SI mixes a classical fingerprint approach with additional information such as the size of the molecule, the presence/absence of heteroatoms and of particular functional groups. It provides a “generic” measurement of structural similarity, taking into account all possible chemical features of the molecules. Here we used an in-house software that employs the SI and can split the molecules of a given data set into chemical similarity-based clusters, in this way the similarity values between molecules inside a cluster is minimized and the similarity values between molecules of different clusters is maximized. The clusters are further grouped into super-clusters, containing all clusters whose average similarity between their corresponding molecules is higher than a given threshold. This further step allows verifying if some produced clusters can be related between themselves for some chemical/toxicological reason. This similarity algorithm relies on a *K*-means approach (in the first step), where an iterative procedure is applied in order to build the most suitable clusters: starting from the initial setting (where each compound represent a cluster for itself) compounds are iteratively moved to the cluster that best maximize the intra-cluster similarity and minimize the similarity between cluster, until no further optimization step is possible. In the second step, the algorithm exploits a hierarchical approach, where clusters are grouped on the basis of a given threshold, to support human expert reasoning (i.e., finding chemical/toxicological issues common to different clusters).

We applied this clustering approach to the positive (hepatotoxic) compounds in the training set (408 compounds) in order to identify SAs only for positive substances. The compounds were automatically divided into 78 clusters with average similarity ranging from 0.677 to 0.980. We checked each cluster and eliminated those with average similarity below 0.7. For each cluster we manually identified a common chemical structure. However, this last step was not possible for every single cluster since the chemicals in the cluster did not always share an unambiguous, unique chemical core. In this case we disregarded the cluster. The chemical cores for each cluster were written as SMARTS (http://www.daylight.com/dayhtml/doc/theory/theory.smarts.html), which is the language that enables to describe the chemical patterns in a more general way than the Simplified Molecular Input Line Entry System (SMILES) (http://www.daylight.com/dayhtml/doc/theory/theory.smiles.html).

**Figure 1:**
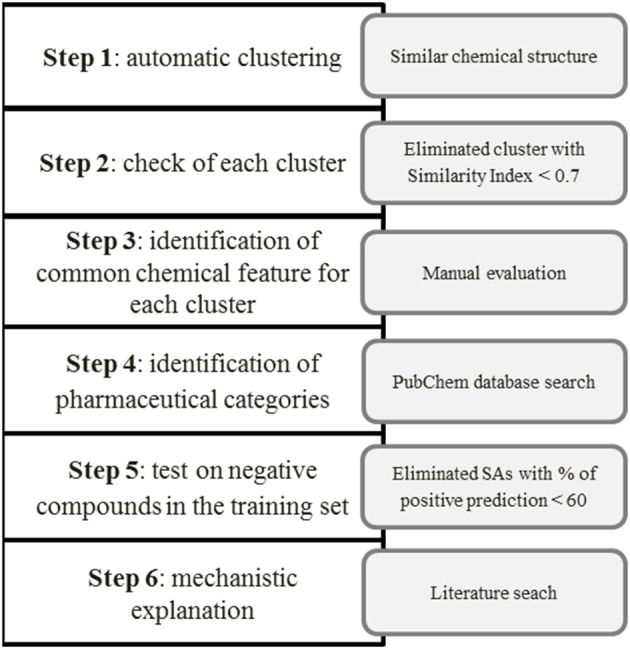
**Identification and validation of SAs for hepatotoxicity**.

The next step was to assign the pharmacological category to each compound (antibiotics, antipsychotic agents, antiviral etc.) using the PubChem database (https://pubchem.ncbi.nlm.nih.gov/)^1^, to clarify whether the chemical similarity is also related to common uses of the drugs. When we considered a matching more appropriate than the one generated automatically by the software, we moved some substances from one cluster to another. Then we tested the SAs identified for the remaining clusters on the negative (non-hepatotoxic) compounds in the training set (352). Finally, we discarded SAs whose percentage of correctly predicted compounds (in terms of true positive, TP or true negative, TN) was below the arbitrarily threshold of 60, in the training set. For each cluster we provided also a mechanistic explanation and/or supporting information in the literature. Figure 1 illustrates the scheme developed for the manual identification of SAs.

#### Automatic extraction of SAs

For the automatic extraction of SAs we used the SARpy software, described in Ferrari et al. (2013). Briefly, SARpy extracts sets of rules by automatically generating and selecting substructures without any *a priori* knowledge, solely on the basis of their prediction performance on a training set used as input. In the first step the input chemicals (training set) are fragmented in order to extract all the substructures within a customizable size range. Then, the software analyses the correlation between the occurrence of each molecular substructure and the experimental activity of the compounds that contain it in the training set. This is a validation step aimed at assessing the predictive power of each fragment. Finally, a subset of fragments is selected and provided to the user in the form of rules “IF fragment THEN activity” (Lombardo et al., 2014). The input and the output of SARpy are chemical structures and sub-structures expressed as SMILES. The statistical parameter used for defining the precision of a fragment to predict the activity under investigation is the likelihood ratio (LR), calculated for each SA as:

$$\begin{array}{l} \text{LR}=\;\frac{\text{TP}}{\text{FP}}\;x\;\frac{\text{negatives}}{\text{positives}} \end{array}$$

Where TP are experimentally positive (toxic) compounds correctly predicted as positive, false positives (FP) are experimentally negative but wrongly predicted as positive and negatives and positives are the number of non-toxic and toxic compounds present in the dataset, respectively.

We ran SARpy on the training set (760 compounds) using different settings (max, min, optimal) as previously described (Lombardo et al., 2014; Pizzo et al., 2015) in order to extract SAs for hepatotoxicity and non-hepatotoxicity. After identifying SAs using manual and automatic approaches, we graphically compared the list of SMARTS of the manually identified SAs and the list of SMILES produced by SARpy. In case of similar SAs that matched the same compounds in the training set, we considered only the manually extracted one and eliminated the other.

#### Performance

Since hepatotoxicity is expressed as a binary classification (hepatotoxic and non-hepatotoxic) we adopted statistical parameters to evaluate the performance such as accuracy, sensitivity, specificity and Matthews correlation coefficient (MCC). To standardize the statistical results, we used the term “positive” to refer to hepatotoxic compounds and “negative” for non-hepatotoxic ones.

Accuracy: this measurement, also known as concordance, gives a general picture of the errors made by the model. It is defined as the ratio of the compounds correctly predicted to the total number of compounds. The result spreads from 0 (no accuracy) and 1 (maximum accuracy).

$$\begin{array}{l} \text{accuracy}=\frac{\left(\text{TP}+\text{TN}\right)}{\left(\text{P}+\text{N}\right)} \end{array}$$

Sensitivity: a model is sensitive when it has good ability to identify true-positives (TP, hepatotoxic compounds correctly classified as hepatotoxic) so few false-negatives (FN, hepatotoxic compounds wrongly classified as non-hepatotoxic) are predicted. It is defined as the ratio of the TP to the total number of positives. The result spreads from 0 (no sensitivity) and 1 (maximum sensitivity).

$$\begin{array}{l} \text{sensitivity}=\frac{\text{TP}}{\left(\text{TP}+\text{FN}\right)} \end{array}$$

Specificity: a model is specific when it has good ability to identify TN, (non-hepatotoxic compounds correctly classified as non-hepatotoxic) so it gives few false positives (FP, non-hepatotoxic compounds wrongly classified as hepatotoxic). It is defined as the ratio of the TN to the total number of negative compounds. The result spreads from 0 (no specificity) and 1 (maximum specificity).

$$\begin{array}{l} \text{specificity}=\frac{\text{TN}}{\left(\text{FP}+\text{TN}\right)} \end{array}$$

MCC: this is a measure of the quality of a binary classification. It considers TN, TP, FN, and FP. The result should be between +1 and −1. If the result is +1, the prediction is perfect, and if it is 0 the result can be considered a random prediction; if the result is −1 there is total disagreement between the predicted and experimental values (Dao et al., 2011).

$$\begin{array}{l} \text{MCC}=\frac{\left(\text{TP}*\text{TN}\right)-\left(\text{FP}*\text{FN}\right)}{\surd \left(\text{TP}+\text{FP}\right)*\left(\text{TP}+\text{FN}\right)*\left(\text{TN}+\text{FP}\right)*\left(\text{TN}+\text{FN}\right)} \end{array}$$

## Results

### Manually extracted SAs

Table 1 illustrates the manually-identified SAs with the total number of occurrences, the number and the percentage of TP in the training set. Performance in test and external validation sets are reported in Supplementary Table 1.

**Table 1:** **Manually extracted structural alerts (SAs) with the total number of occurrences and the number and percentage of true positive (TP) in the training set**.

**ID**	**SMARTS**	**Activity**	**Chemical structures**	**Pharmacological class**	**Total occurrences in the training set**	**N. of TP (%TP)**	**N. FP (%FP)**
1	[n,c]1ccn[n,c]c1	Hepatotoxic	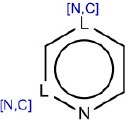	N-containing heterocycles aromatic compounds (pyridine, pyrazine, pyrimidine)	57	41 (71.40)	16 (28.60)
2	NS(= O)(= O)c1ccccc1	Hepatotoxic	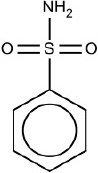	Sulphonamides	31	22 (70.96)	9 (29.04)
3	OC(= O)C1[C,S][S,O,C]C2CC(= O)N12	Hepatotoxic	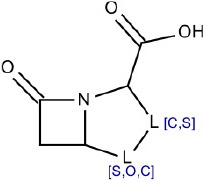	β-lactam antibiotics (penicillin)	12	8 (66.66)	4 (33.34)
4	O = C1N~CC = C[N,C]1C2C~[S,C]CO2	Hepatotoxic	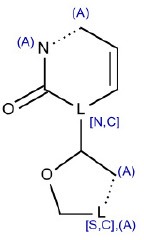	Nucleoside analogs	11	9 (81.80)	2 (18.20)
5	C1[S,C,N,O]c2ccccc2[N,C,S,O]c3ccccc13	Hepatotoxic	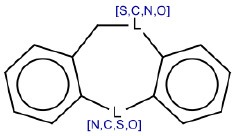	Tricyclic antidepressants (TCAs)	11	9 (81.80)	2 (18.20)
6	[N;!$([N+]);!$(NC = O);!$(N = [N,C,O])][a]	Hepatotoxic	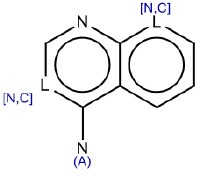	Aromatic amines	10	6 (60.00)	4 (40.00)
7	O = C1CCCCCCCCCCCCO1	Hepatotoxic	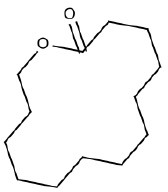	Macrolide antibiotics	7	5 (71.40)	2 (28.60)
8	Nc1[n,c]cc2C(= O)C(= CNc2[c,n]1)C(O) = O	Hepatotoxic	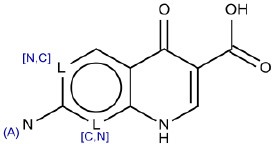	Anti-bacterial agents (fluorquinolone)	6	4 (66.66)	2 (33.34)
9	^*^N(^*^)CCC(c1cccc[n,c]1)c2cccc[n,c]2	Hepatotoxic	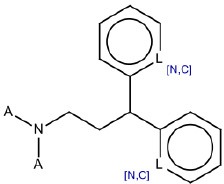	Cationic amphiphilic drugs (CADs)	6	5 (83.33)	1 (16.67)
10	CC = C(C)C = CC = C(C)C = C[R,a]	Hepatotoxic	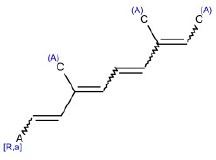	Retinoids	4	3 (75.00)	1 (25.00)
11	CNC(= O)N(CCCl)N = O	Hepatotoxic	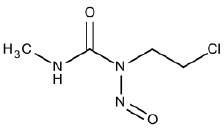	Nitrosourea compounds	2	2 (100)	0 (0)
12	C1CC2CCC3C(CC[C,c]4[C,c][C,c][C,c][C,c][C,c]34)C2C1	non-hepatotoxic	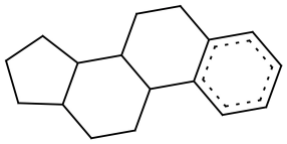	Steroids	23	16 (TN) (69.56)	7 (FN) (30.44)
13	CC(= O)NC1C2[S,O]CC = C(N2C1 = O)C(O) = O	non-hepatotoxic	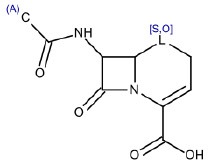	β-lactam antibiotics (cephalosporins)	16	11 (TN) (68.75)	5 (FN) (31.25)

We used Marvin for drawing and displaying chemical structures and substructures (Marvin 5.11.5, 2013, ChemAxon) (http://www.chemaxon.com)^2^.

Totally, we identified 13 SAs, 11 of them considered hepatotoxic.

SA identified with ID 1 (N-containing heterocycles aromatic compounds (pyridine, pyrazine, pyrimidine) matched the highest number of compounds in the training (57), test (19) and external validation (9) sets. Its performance in the training and in the test sets was high (71.4 and 88.9 respectively).

SA with ID 2 (sulphonamides) performed well in all three sets of compounds reporting TP% of 70.96, 75, and 83 in the training, test and external validation sets respectively.

SA identified by ID 3 (β-lactam antibiotics, penicillin) reported TP% of 66.66 in the training set and of 100 in the test set. It did not match any chemicals in the external validation set.

SAs with ID 4 (nucleoside analogs) and 5 (tricyclic antidepressants, TCAs) reported both TP% of 81.8% in the training set. SA with ID 4 did not perform well in the test set (TP% 50) but had good performance in the external validation set (TP% 100), on the contrary SA with ID 5 reported TP% of 100% in the test set, but it did not match any compound in the external validation set.

SA with ID 6 (aromatic amines) had poor performance in the training set (TP% 60) however, in test and external validation sets it reported 100% of TP.

SA with ID 7 (macrolide antibiotics) had good performance in training and test sets (TP% 71.4 and 100 respectively), however in the external validation set it did not match any compound.

SA with ID 8 (anti-bacterial agents, fluorquinolone) had poor performance in the training set (TP% 66) however, in both test and external validation sets it reported TP% of 100.

SA identified with ID 9 (cationic amphiphilic drugs, CADs), reported good performance in the training set (TP% 83.33). In the test and external validation sets it did not match any compounds.

SA identified with ID 10 (retinoids) had good performance in the training and test sets (TP% 75 and 100 respectively), and it did not match any compound in the external validation set.

Similarly, SAs identified with ID 11 (nitrosourea compounds) reported good performance in the training set with TP% of 100, however in the test and external validation sets they did not match any compounds.

Although we only used positive compounds to extract SAs, we labeled the SAs identified by ID 12 and 13 as non-hepatotoxic since they matched more experimentally non-hepatotoxic compounds than hepatotoxic ones (Figure 1, step 5). Both SAs gave TN% close to 70 in the training set.

SA with ID 12 (steroids) had bad performance in the test (TN% 33), but in the validation set it identified correctly negative compounds (TN% 100). On the contrary,

SA identified with ID 13 (β-lactam antibiotics, cephalosporins) performed well in the test set reporting TN% of 75, but it did not match any compounds in the external validation set.

### Automatically extracted SAs

Using SARpy software, we were able to identify 75 SAs, 40 of them related to hepatotoxicity. Once generated by SARpy, each SA was carefully checked. In order to keep only the reliable SAs, we deleted those with percentages of TP below the arbitrary threshold of 70. For SA with ID 40 we generalized the original SMARTS in order to get a new one that correctly matched more compounds than the original one.

The complete list of SAs for hepatotoxicity and non-hepatotoxicity is available in Supplementary Table 2; the statistical performance of each SA, in terms of total number of occurrences and the number and percentage of TP in the training, test and external validation sets are also provided. Due to the relative high number of SAs extracted with SARpy software compared to the number of molecules available for the test and external validation sets, the total occurrences of 34 and 37 out of 75 SAs were null in the test and external validation set, respectively.

SAs related to hepatotoxicity with ID 7, 8, 9, and 29 reported 100% of TP in training, test and external validation sets. On the contrary SA with ID 3 and ID 6 had good performance in training set (TP % 100), but in the test and external validation sets they did not match any compound. SA with ID 36 and 38 had good performances in the training set (TP % of 71.42 and 70.96, respectively) while their performance increased in the test (100 and 75.00% of TP, respectively) and external validation set (TP % of 100 and 83.33, respectively).

SA with ID 41 identified correctly negative compounds (TN % of 100) in the training, test and external validation sets. SAs with ID 44, 47, and 57 had good performance in the training and test sets (TN % of 100); unfortunately their performance in the external validation set was not evaluated since their occurrences were null. SA with ID 66 performed well in the training set (TN % of 83.33) and in the test and external validation sets it identified correctly negative compounds (TN % 100). SAs with ID 49, 50, 51, 54, and 63 reported 100% of TN in the training set but in the test and/or external validation set it did not match any compound.

### Decision tree

**Figure 2:**
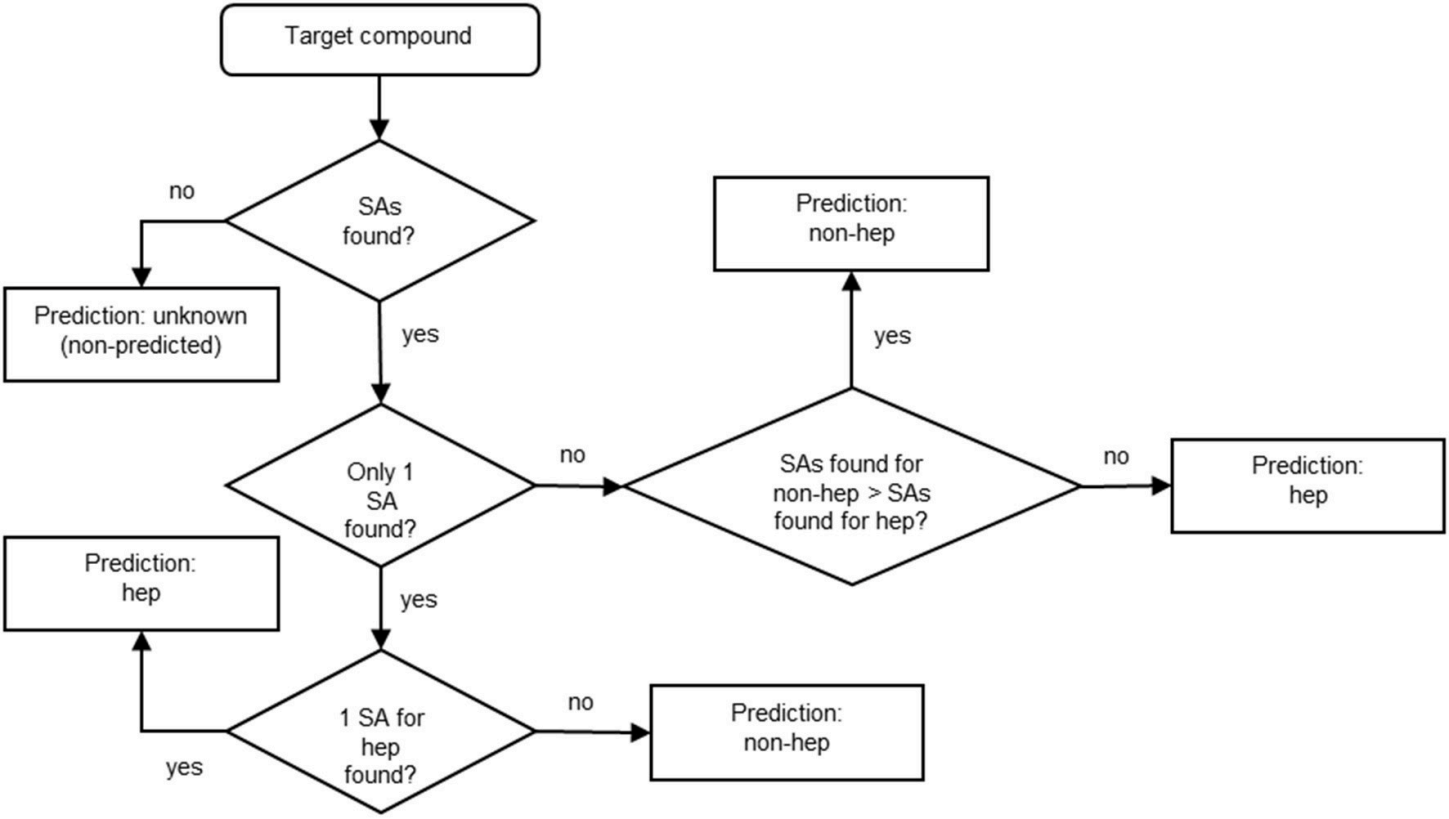
**Decision tree developed for the hepatotoxicity model**. Hep stands for “hepatotoxic” and non-hep for “non-hepatotoxic.”

After identifying the SAs, we established a reasonable strategy for manually building the model basing on the expert-based knowledge. Figure 2 shows the decision tree we applied for building the model for the prediction of hepatotoxicity. Basically, if no SAs are found for the target compound, no prediction is provided and the compound is labeled “unknown (non-predicted).” If one SA is identified, the prediction for the target compound is hepatotoxic or non-hepatotoxic depending on the SA. If more than one SAs is found, the prediction depends on the number of SAs: if more SAs for non-hepatotoxicity are found than those for hepatotoxicity, the target compound is predicted as non-hepatotoxic; otherwise (the number of SAs found for non-hepatotoxicity is lower or equal to the number of SAs found for hepatotoxicity) it is hepatotoxic. Since it is preferable to overestimate hepatotoxicity rather than not to recognize unsafe compounds, the overall model's architecture followed a conservative approach.

### Results on the training, test and external validation sets

**Figure 3:**
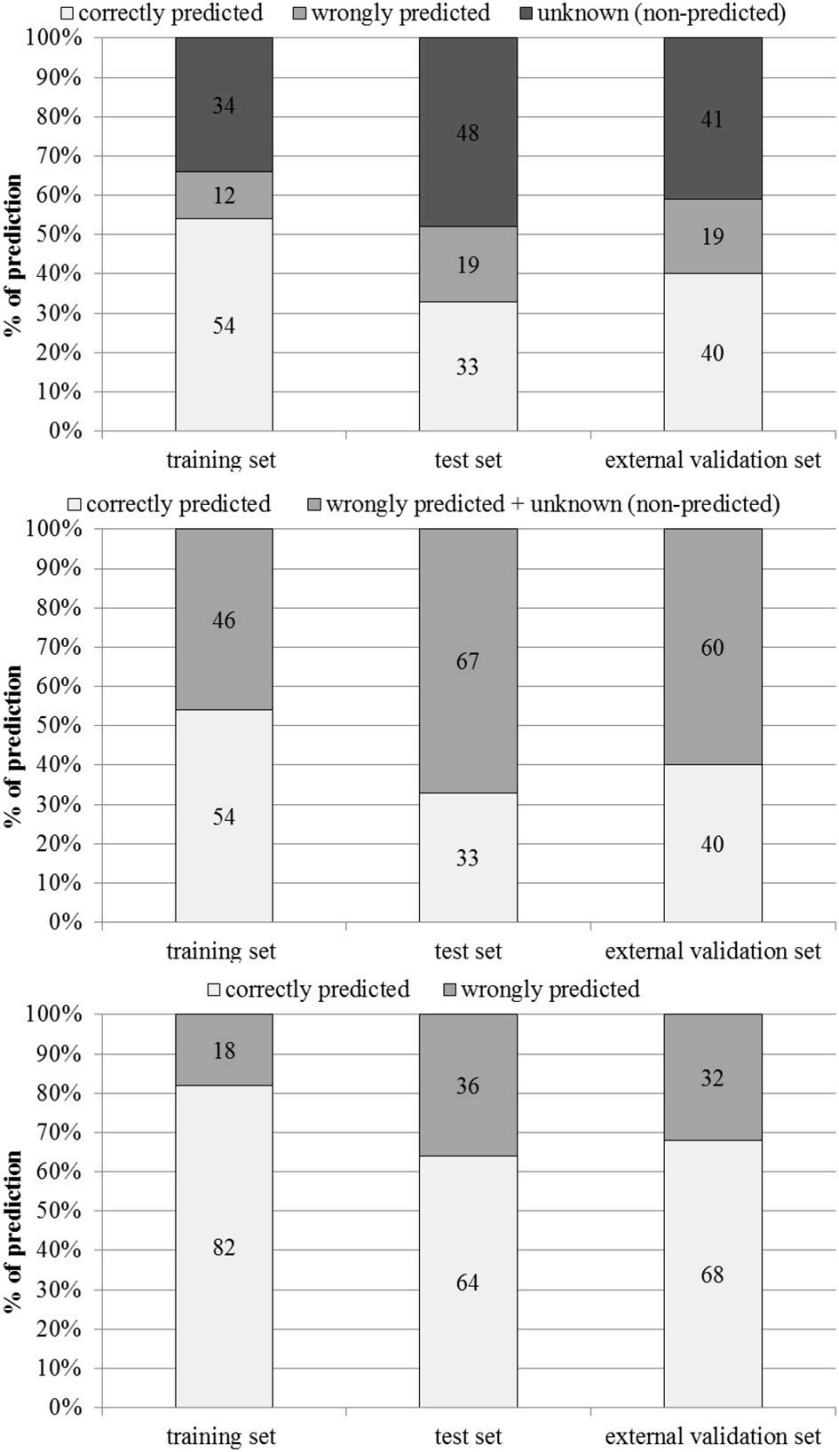
**Percentages of correctly predicted, wrongly predicted and non-predicted (unknown) compounds in the training, test and external validation sets**.

The performance of the model in the training, test and external validation sets is illustrated in Figure 3 and Table 2.

**Table 2:** **Performance of the model in the training, test and external validation sets**.

	**Training set**	**Test set**	**External validation set**
Number of compounds	760	190	101
Number of TP	263	48	35
Number of FP	72	30	10
Number of TN	144	15	5
Number of FN	18	6	9
Number predicted	497	99	59
Number unknown	263	91	42
Accuracy	81	63	68
Sensitivity	93	88	80
Specificity	67	33	33
MCC	0.64	0.27	0.13

Out of 760 compounds that were present in the training set, 263 were not predicted by the model (unknown, non-predicted). 263 compounds were correctly predicted as hepatotoxic (TP) and 144 were correctly predicted as non-hepatotoxic (TN). 72 molecules experimentally non-hepatotoxic were identified by the model as hepatotoxic (FP) and only 18 compounds experimentally hepatotoxic were predicted as non-hepatotoxic (FN). For 91 compounds in the test set (190 molecules) the model did not provide any prediction (unknown, non-predicted), 48 compounds were correctly identified as hepatotoxic (TP) and 15 as non-hepatotoxic (TN). The number of experimentally negative (non-hepatotoxic) compounds wrongly predicted as hepatotoxic (FP) was 30 and the number of positive compounds (hepatotoxic) wrongly predicted as negative (FN) was 6. In the external validation set (101 compounds), 59 chemicals were not predicted by the model (unknown, non-predicted), the numbers of TP and TN was 35 and 5 respectively. 10 compounds were wrongly classified as hepatotoxic (FP) and 9 as non-hepatotoxic (FN).

Performance in the training set was good on all four parameters (accuracy 81%, sensitivity 93%, specificity 67 and MCC 0.64). As expected, in the test and external validation sets the statistical parameters tended to decrease, mainly specificity (33% in both test and external validation sets). However, in the test and external validation sets accuracy (63% and 68%), sensitivity (88% and 80%) and MCC (0.27 and 0.13%) were satisfactory.

Figure 3 shows percentages of correctly predicted and wrongly predicted compounds in the training, test and external validation sets. In the training set 54% of the compounds were correctly predicted and the prediction was wrong only for 12%. The model did not provide any prediction for 35, 48, and 41% of the substances present respectively in the training, test and external validation sets. However, excluding the non-predicted compounds, the percentages of correct prediction were 82, 64, and 68% in the training, test and external validation sets.

## Discussion

### Limitations and weaknesses of experimental hepatotoxicity data

High-quality and reliable biological data are essential in order to build predictive models to provide relevant information about the toxicological behavior of a substance. Ideally the data for building a model should be obtained using a unique, well-standardized protocol, in the same laboratory by the same scientists. It is also important that these data refer to a clear and unambiguous endpoint (Cronin and Schultz, 2003). However, this is difficult, especially for hepatotoxicity, since the data are spread out in the literature and databases, refer to several endpoints related to hepatotoxicity (steatosis, colestasis, fibrosis etc.) and are obtained with different laboratory methods. Then, as previously mentioned, there is no a good single standard indicator of DILI with high sensitivity and specificity (Przybylak and Cronin, 2012). Indeed, no well-defined biomarkers exist for the identification of hepatotoxicity *in vitro* or *in vivo*. Consequently, the data in the literature refer to different effects and mechanisms of action underlying the endpoint of hepatotoxicity. Here we used one of the largest data sets available for DILI (Fourches et al., 2010). This data set, compiled using the data mining procedure, suffers some limitations. Firstly it does not make any distinction between idiosyncratic and dose-dependent toxicity. Idiosyncratic toxicity refers to an abnormal reaction to a drug that is not connected to its pharmacological activity but is due to individual hypersensitivity (Cheng and Dixon, 2003; Russmann et al., 2009). This toxicity does not follow any specific mode of action, but the adverse reactions to drugs are of unknown etiology and involved only a small proportion of the population (Walgren et al., 2005). Furthermore, in this data set, the compounds labeled as “negative” do not refer to compounds without reported effects, but to those without information in the literature. This means that where information is lacking it has been assumed that the compound was negative. Even if it is true that for well-known and investigated drugs, the lack of information can be taken as negative (Hewitt et al., 2013) this can lead to a large amount of FN in the data set that can in the end interfere with the data modeling.

Concerning the second data set we used, the US FDA Human Liver Adverse Effects Database, other limitations need to be discussed. This data set classifies compounds as active (hepatotoxic) and inactive (non-hepatotoxic) on the basis of reported alterations for five hepatic enzymes (ALP, AST, ALT, LDH and GGT). When the hepatocyte membrane is damaged these enzymes, which are normally located in the cytosol, are released into the bloodstream (Pari and Murugan, 2004). Although the serum transaminases are commonly used as indicators of liver injury and reflect damage to hepatocytes (Ozer et al., 2008), they are not always reliable and specific for the detection of hepatotoxicity. For example, ALT and AST are present in other tissues (heart, brain and skeletal muscle) besides the liver and so they are released into the circulation when there is damage to these tissues. AST mostly increases in case of myocyte damage due to extreme physical effort (Ozer et al., 2008). LDH is another enzyme occasionally used as a biomarker of hepatocellular injury. However, it is not routinely employed since its specificity is questionable (Ramaiah, 2007). More recently genomics, proteomics and metabolomics have been proposed as valuable techniques for discovering biomarkers (Amacher et al., 2005) and gene-expression profiling and microRNAs as more sensitive and specific indicators of DILI (Blomme et al., 2009; Laterza et al., 2009). Despite its limitations, however, in our opinion the US FDA Human Liver Adverse Effects Database is a good choice for modeling since the source (US FDA) is reliable and the results are based on objective laboratory parameters (serum transaminases). However, the most of the datasets is not suitable to be used alone for classification modeling. In conclusion, the data we used for modeling have a certain level of uncertainty due to these points which may have influenced the reliability and performance of the model.

An alternative that could limit the uncertainty linked to hepatotoxicity data is to use *in vitro* data obtained if possible on the same cell lines and using the same laboratory assay and conditions. However, not much public data is available in the open literature for this purpose and this approach too suffers some limitations such as the influence of genetic and environmental factors in the variations of biochemistry (Przybylak and Cronin, 2012).

### Mechanistic explanation of SAs

We propose, when possible, a mechanistic rationale using the information in the literature and in public databases (PubChem https://pubchem.ncbi.nlm.nih.gov/, LiverTox, http://livertox.nih.gov/) for each SA that we manually identified through the chemical category approach.

#### SA ID 1: N-containing heterocyclic aromatic compounds: pyridine, pyrazine, pyrimidine

This SA, identified by the ID number 1 (Table 1), is a generic chemical structure that may be seen in several different chemical families. In the training set it matches 57 compounds (covering different chemical and therapeutic classes), 41 of them classified as hepatotoxic. Considering the lack of specificity of this SA, it is impossible to highlight any single mechanism of action that may explain the toxicity. In the training set this chemical fragment correctly identified hepatotoxic compounds in 71.4% of cases and that is enough to retain this alert. However, we identified two sub-families in this large group of drugs. The first comprises a group of three drugs used for the treatment of malaria (amodiaquin, primaquine, mefloquine). The hepatotoxicity of these molecules are mainly linked to hypersensitivity reactions (LiverTox database, http://livertox.nih.gov/).

The second includes seven chemical compounds used in the therapy of several cancer types (methotrexate, amsacrine, bortezomib, imatinib mesilate, intoplicine, OSI-461, rubitecan).

Methotrexate is a methyl analog of folic acid, used in the treatment of various neoplastic diseases. A reasonable part of the population treated with this drug reported liver injury. The typical histologic features of methotrexate toxicity are aspecific and comprise steatosis, “glycogen” nuclei, multinucleation, anisonucleosis, and lipofuscin accumulation, chronic inflammation of portal tracts, bile duct damage, ductular reaction, and fibrosis. Thin fibrous septa extending from the portal tracts into the lobules, often in a stellate configuration is the typical pattern of liver fibrosis induced by methotrexate. Persistent fibrosis eventually may lead to cirrhosis (Hytiroglou et al., 2004).

The mechanisms of liver injury of bortezomib are still unclear. It is metabolized in the liver largely through the CYP 3A4 pathway and liver injury may be related to production of a toxic intermediate (LiverTox database).

Therapy with imatinib may lead to three forms of acute liver injury: transient and usually asymptomatic elevations in serum enzymes during treatment, clinically apparent acute hepatitis, and reactivation of an underlying chronic hepatitis B (LiverTox database).

Metabolism of OSI-461 occurs in liver and preclinical repeated dose toxicity studies reported liver injury at higher doses. In a recent study (O'Bryant et al., 2009), treatment OSI-461 caused only mild to moderate and reversible transaminase and bilirubin increase.

#### SAs ID 2: sulphonamides

This SA identified by the ID number 2 (Table 1) matches chemicals containing a sulfonamide group in their structures. Most of the compounds correctly predicted as hepatotoxic by this SA are sulfonamide antibiotics. Several compounds belonging to this class have been reported to cause mild cholestasis hepatitis, but severe and even fatal cases have occurred (Polson, 2007). Sulfonamide antibiotics are among the most common causes of allergic or hypersensitivity reactions and it has been estimated that 6% of patients treated with this class of antibiotics have shown an immune-mediated event. The percentage rises steeply (60%) for HIV-positive individuals (Brackett, 2007). From a histopathological point of view centrilobular cholestasis is frequent in sulfonamide hepatotoxicity with only mild to moderate mixed portal inflammation of lymphocytes, and small numbers of eosinophils and neutrophils (Murray et al., 2008). Granulomatous hepatitis is uncommon but it can occur with many of the sulfonamides while massive hepatocellular necrosis has been described in fatal cases (Murray et al., 2008).

#### SA ID3: β-lactam antibiotics (penicillin)

The β-lactam ring, which is shared by many penicillin-like antibiotics, represents the SA identified with ID number 3 (Table 1). This SA matched 12 compounds in the training set, eight of them are labeled as hepatotoxic. Six are β-lactam antibiotics (azlocillin, carbenicillin, amoxicillin, flucloxacillin, oxacillin and penicillin) with the core structure of penicillins and two are used as β-lactamase inhibitors (clavulanic acid and sulbactam). β-lactams have been associated with small increases in serum enzymes (Zimmerman, 1999). However, more severe liver diseases, such as hepatitis and or intrahepatic cholestasis, have been reported: β-lactam and the isoxazolyl penicillins (oxacillin) are the most frequently involved (Olans and Weiner, 1976). Amoxicillin which is normally used in combination with clavulanic acid in order to reduce antibiotic-resistance can cause from mild to moderate hepatitis that rarely leads to liver failure. The mechanism of action that leads to toxicity is still not completely clear. However, several human leukocyte antigen haplotypes have been found to be related with hepatotoxicity, especially in the elderly (Pugh et al., 2009). A few severe reactions, including bile duct damage have been reported (Cundiff and Joe, 2007). Prolonged treatment with flucloxacillin in the elderly has been associated with jaundice (Fairley et al., 1993), and cholestatic liver injury has been described, most often with flucloxacillin (Devereaux et al., 1995).

#### SA ID 4: nucleoside analogs

This SA, marked with the ID number 4 (Table 1), identifies two classes of compounds in the training set: the antiretrovirals (nucleoside analogs reverse transcriptase inhibitors, NRTI) and anti-cancer nucleoside drugs. Four compounds are found in the first class: lamivudine, stavudine, zidovudine and 2′-fluoro-5-methyl arabinosyl uracil, all used for the treatment of HIV or hepatitis B infection. Some cases of liver injury have been reported in patients taking zidovudine, but stavudine is responsible for more severe hepatotoxicity (Nunez, 2006).

The second class of drugs matched by the SA with ID 8 is nucleoside analogs used for the treatment of malignancies (cytarabine, capecitabine, doxifluridine, uridine, and 5-fluoro-2′-deoxyuridine). They are a family of drugs that inhibit DNA synthesis either directly or through inhibition of DNA precursor synthesis (Diab et al., 2007). Several NRTI induce hepatotoxicity through mitochondrial damage (Nunez, 2006). The mechanisms of this liver damage is explained well in Boelsterli and Lim (2007) and Dykens and Will (2007). Briefly, NRTI inhibit the polymerase that replicates mitochondrial DNA, preventing mitochondrial replication and finally leading to a reduction in mitochondrial function in several tissues (liver and muscle toxicity), and also lipodystrophy and lipoatrophy (Dykens and Will, 2007).

Due to their lack of selectivity toward tumor cells, nucleoside analogs are cytotoxic interfering with the physiological cellular metabolism and deregulating the nucleoside/nucleotide pools in both normal and cancerous cells. These drugs cause several side effects such as myelosuppression, hepatotoxicity, renal toxicity, leucopenia, thrombocytopenia and mucositis (Diab et al., 2007).

#### SA ID 5: triciclycic antidepressants (TCAs)

We identified the SA with ID 5 (Table 1) starting from nine compounds in the training set and labeled as hepatotoxic. Among these nine, seven are triciclycic antidepressants (TCAs), one is also an antidepressant but with four rings (mianserin) and one is an antihistamine with a tricyclic group (cyproheptadine).

TCAs, developed in the 1950s, are a group of compounds that share similar chemical structures and have antidepressant potential in humans. The use of these drugs as antidepressants dropped steeply with the introduction of selective serotonin-reuptake inhibitors and other new-generation molecules. However, they are still used for several off-label prescriptions. Their decrease has meant that few hepatotoxicity cases have been reported in the past 15 years (DeSanty and Amabile, 2007). However, TCAs have been associated with hepatotoxic and cholestatic reactions (Ilan et al., 1996; de Abajo et al., 2004). Asymptomatic increases in transaminase serum levels are a common side effects with this group of drugs (Price et al., 1983; Ramesh et al., 1990) and severe hepatitis and acute liver failure cases are also reported (Lucena et al., 2003).

As mentioned, this group of drugs shares a similar chemical structure so cross-hepatotoxicity is possible (Larrey et al., 1986; Remy et al., 1995). Some TCA-induced hepatotoxic reactions seemed to be immune-mediated since significant extrahepatic symptoms, eosinophilia, and eosinophilic infiltration of the liver have been reported (Anderson and Henrikson, 1978). Hypothetically TCAs could show the same mechanism of action (cholestatic injury) as chlorpromazine since these compounds present structural similarity (Selim and Kaplowitz, 1999; de Abajo et al., 2004).

#### SA ID 6: aromatic amines

We identified ten molecules in the training set containing the SA with ID 6 (Table 1), six of them were labeled as hepatototoxic. Three compounds (amsacrine, gefinitib and vandetanib) are antineoplastic agents used for the treatment of several cancer types. The others are one antimalarial agent (amodiaquine), a non-narcotic analgesic (glafenine) and an α-adrenergic blocker (prazosin). The common chemical feature is the aromatic amine. Although no specific mechanism of action for the liver toxicity is described, *in vivo* studies on animal models indicated that aromatic amines exert liver toxicity by inducing cellular oxidative stress (Hillesheim et al., 1995; Ambs and Neumann, 1996).

#### SA ID 7: macrolide antibiotics

The SA identified with the ID number 7 (Table 1) correctly identified five hepatotoxic compounds out of the seven chemicals matched. These five are all macrolide antibiotics. The main member of this class is erythromycin. Hepatic dysfunction after treatment with these drugs is occasional. Erythromycin estolate has been associated with cholestatic hepatitis in particular (Hashisaki, 1995). Through their hepatic metabolism, reactive oxygen species (ROS), such as superoxide anion (O${}_{2}^{-}$) and hydrogen peroxide (H_2_O_2_), are created, leading to the production of free radicals such as OH^•^. These hydrogen species bind polyunsaturated fatty acids starting the lipid peroxidation that causes oxidative degradation and inactivation of biomolecules (Pari and Murugan, 2004).

#### SA ID 8: anti-bacterial agents (fluoroquinolone)

The SA marked with the ID number 8 (Table 1) matched six compounds in the training set, four of them are hepatotoxic. The chemicals correctly identified as hepatotoxic belong to the fluorquinolones class of antibiotics. These are anti-bacterial agents are widely prescribed and used (Orman et al., 2011). Side effects from fluorquinolones are uncommon, though significant adverse effects with this drug category have been reported in the gastrointestinal tract, the central nervous system (CNS), heart, cartilaginous tissues and skin (Stahlmann and Lode, 1999). Numerous cases of severe liver injury were reported only for trovafloxacin that was subsequently withdrawn from the market (Ball et al., 1999; Orman et al., 2011).

Concerning the other members of the drugs family, there are occasional case reports of hepatotoxicity (Orman et al., 2011). However, one investigation (Paterson et al., 2012) reported 88 fatal cases of acute liver injury among 144 patients in hospital after treatment with fluorquinolone and with no evidence of preexisting liver disease.

#### SA ID 9: cationic amphiphilic drugs (CADs)

The SA with ID number 9 (Table 1) comprises a group of heterogeneous drugs and matched six compounds in the training set, five of which were correctly identified as hepatotoxic (chlorpheniramine, disopyramide, doxapam, methadone, and tolterodine). From a chemical point of view this structure is a typical cationic amphiphilic since it has a hydrophobic ring structure and a hydrophilic side chain with an amino group. With the exception of doxapram, all the other drugs matched by SA number 9 are CADs. This category is known to have the potential to cause phospholipidosis which involves excessive accumulation of phospholipids within cells (Sawada et al., 2005). Any tissue can be potentially affected by phospholipidosis and excessive accumulation is commonly found in the lung, liver, brain, kidney, ocular tissues, heart, adrenal glands, hematopoietic tissue, and circulating lymphocytes (Halliwell, 1997). It has been reported that CADs induce phospholipidosis by inhibiting lysosomal phospholipase activity; however the specific mechanism is still not clear (Sawada et al., 2005).

#### SA ID 10: retinoids

This SA, identified with the ID number 10 (Table 1), is the typical carbon chain of retinoids. It matches four compounds, three of them are retinoids (etretinate, vitamin A, fenretidine), labeled as hepatotoxic and one, a terpene (astaxanthin), as non-hepatotoxic. It is well-known that excessive intake of retinol (vitamin A) is toxic. Hepatomegaly and cirrhosis have been reported in patients given retinol (Myhre et al., 2003). The toxicity of vitamin A is reviewed in Penniston and Tanumihardjo (2006). The mechanism of toxicity of retinoid derivatives has not yet been clarified, but they may alter glycoprotein synthesis and/or genomic expression, inducing membrane injury in hepatic cells (Fallon and Boyer, 1990).

#### SA ID 11: nitrosourea compounds

The SA identified by ID number 11 (Table 1) comprises the so-called nitrosourea compounds. We identified it using two compounds in our data set: carmustine and lomustine. Both are alkylating agents used in the therapeutic protocols for many types of cancer. Their hepatotoxic effect is usually transient and is associated with glutathione depletion, which that leads to oxidative injury (King and Perry, 2001; Sümbül and Özyilkan, 2010). These compounds have also been associated with rises in aminotransferase levels, with rare fatalities (Thatishetty et al., 2013).

#### SA ID 12: steroids

We identified 23 molecules that contain the SA with ID 12 (Table 1). Only in seven cases the molecules in the training set were labeled as hepatotoxic (ursodiol, 2-methoxy estradiol, betulinic acid, estrone, ethynil estradiol, ethinyl estradiol 3-methyl-ether, oxymethodone), so we retained this SA for identifying non-hepatotoxic compounds. Among the compounds labeled as non-hepatotoxic we found three chemicals belonging to the class of steroidal neuromuscular-blocking drugs (pipecuronium, rocuronium and vecuronium) which are mainly employed in anesthetic practice (Sparr et al., 2001). To the best of our knowledge the side effects reported for this class of compounds relate to the cardiac vagus nerve that leads to cardiovascular effects (Larijani et al., 1989; Sparr et al., 2001). Another group of compounds are digitoxin and digoxin that belong to the class of cardiac glycosides, used as cardiotonic (Fabricant and Farnsworth, 2001). No studies reported hepatotoxicity after treatment with these compounds. A recent investigation (Rabadia et al., 2014) indicated that digitoxin and digoxin had hepato-protective activity in albino rats exposed to carbon tetrachloride (CCl4).

Dehydrocholic acid is also present among the compounds labeled as non-hepatotoxic; and in fact this chemical is hepato-protective (Herraez et al., 2009). The other compounds identified by the SA with ID 14 are mainly estrogens (estradiol derivatives). In some cases these are labeled as hepatotoxic and in others non-hepatotoxic. This may depend on the different substitutions that somehow affect their toxicity.

#### SA ID 13: β-lactam antibiotics (cephalosporins)

The SA identified with the ID number 13 (Table 1) matches 16 compounds in the training set. Only five were labeled as hepatotoxic so we decided to retain this SA for identifying negative (non-hepatotoxic) compounds. The non-hepatotoxic compounds matched by this SA belong to the class of cephalosporin antibiotics. The cephalosporins are a safer class of antibiotics than other commonly used antibiotics such as aminoglycosides, sulfonamides, tetracyclines, and penicillins (SA ID 4) (Neu, 1990). From a chemical point of view, both penicillin and cephalosporin antibiotics have a four-member β-lactam ring, but cephalosporins have a six-member dihydrothiazide ring in place of the five-member thiazolidine ring of penicillin (Ong, 2014). Cephalosporins generally cause few side effects and seem less allergenic than the molecules belonging to the penicillin group (Neu, 1990; Dancer, 2001). They rarely cause idiosyncratic hepatotoxicity (Pugh et al., 2009). Since cephalosporins are mostly excreted in the urine, the main toxicity is related to the kidney where they can cause dose-related nephrotoxicity and hypersensitivity interstitial nephritis (Maher, 2013).

### Comparison with other *in silico* models for the prediction of hepatotoxicity and general considerations

Since the prediction of toxicity at organ level is a recent acquisition, so far only a small number of *in silico* models are available for hepatotoxicity. These models differ considering both the data used (*in vitro* or *in vivo*, human or animal models) and the techniques applied (statistical or expert-based). *In silico* models for the prediction of hepatotoxicity have been reviewed (Cheng and Dixon, 2003; Przybylak and Cronin, 2012). To the best of our knowledge, the only comparison with our model is the technique developed by Greene et al. (2010), who starting from a data set of 1266 compounds, identified 38 SAs related to hepatotoxicity. These SAs were implemented into the commercial software Derek for Windows, developed by Lhasa Limited, and the predictive ability of the model was tested on an external data set (626 compounds). In terms of accuracy this model gave lower performance (56%) in the test set than the one we developed (63% in the test set and 68 in the external validation set). Sensitivity was lower too: 46 vs. 88% (test set) and 80% (external validation set), while specificity was higher for Greene's model (73 vs. 33% of our model). In our model the sensitivity is high because the small number of FN in the training, test and external validation sets: 18, 6, and 9 respectively corresponding to 2.4, 3.1, and 8.9% of the total number of compounds in the three sets. In contrast, the number of FP is high (72, 30, and 10 respectively in the training test and external validation sets) leading to a loss of performance in terms of specificity. However, the poor specificity mainly in the test and external validation sets can be explained by two different aspects. Firstly, the conservative approach we followed. Indeed, since it is preferable to overestimate hepatotoxicity rather than not to recognize unsafe compounds, the model followed a conservative architecture. When of a compound is matched by more than one SA and the SAs do not agree (one related to hepatotoxicity and another to non-hepatotoxicity) the final prediction is for positive activity, hence hepatotoxicity. This approach may lead to more of FP, in other words non-hepatotoxic compounds wrongly predicted as hepatotoxic influence the performance of the model, mainly for the specificity. Secondly, the SAs extracted for the negative property performed worst compared to those extracted for the positive activity, leading to a decrease in the ability of the model to correctly identify non-toxic compounds. This may be due to the uncertainty mainly of the negative data as already discussed in Section Limitations and Weaknesses of Experimental Hepatotoxicity Data.

The high number of FP in the present model may have an impact also in the context of drug developmental process. However, two aspects should be taken into account. Firstly, the *in silico* models should not considered as “black box,” but the expert-based judgment is needed to correctly interpret the model output. Secondly, *in silico* models should be used in the framework of an integrated testing strategy, in support and addition to other techniques.

Besides the final performance and the availability of the models (commercial for Derek for Windows but free for our model), there are other differences between the two *in silico* models. One of the main differences from our model is that the SAs implemented into Derek for Windows refer only to hepatotoxicity. This means that if a SA related to hepatotoxicity is identified for a compound, it is predicted as positive (hepatotoxic) otherwise a prediction is not provided by the model. In our model we identified specific SAs for non-hepatotoxicity that are used for the final prediction. If no alerts are identified for a compound, it is not predicted, so is classified as “unknown.” The absence of a specific SA for a compound is not enough proof to classify the compound as negative, hence safe (Przybylak and Cronin, 2012). The identification of SAs for negative activity is a novel approach. To the best of our knowledge, most of the *in silico* models based on SAs can predict the non-toxicity of a substance only on the basis of a lack of information for toxicity. However, an exception related to human toxicity exists, such as the model for mutagenicity developed by Ferrari et al. (2013).

Another model has been developed for the prediction of hepatotoxicity, described in the literature and based on SAs (Egan et al., 2004). Starting from a training set of 244 compounds, most of which were drugs, 74 SAs were identified for hepatotoxicity. However, no information was reported on the statistical performance of the SAs in the training and test sets, so it is hard to make any comparison on the overall application.

Hewitt et al. (2013) using the Fourches et al. (2010) data set, did not build up a real model but identified a list of 16 SAs for hepatotoxicity using the chemical categories approach. Some of these SAs are the same as those we found. In particular, Hewitt et al. (2013) generated sulfonamides SA (ID number 2) and retonoids SA (ID number 10). They also found steroids derivatives SA (ID number 12) and β-lactam antibiotics SAs (ID numbers 3 and 13), but they were associated to the prediction of hepatotoxicity. We used the same SAs (SA 12 and SA 13) for the prediction of non-hepatotoxicity of compounds, since we distinguished for β-lactam antibiotic SAs between penicillins (labeled as hepatotoxic) and cephalosporins (labeled as non-hepatotoxic). The differences between Hewitt et al. (2013) findings and our may be explained considering that we used the Fourches et al. (2010) data set in addition to the US FDA data set. We also extracted the SAs starting from the positive compounds present in the training set and not on the whole data set as the other authors did. Moreover Hewitt et al. (2013) accepted a threshold of similarity lower than to the one we applied (0.6 vs. 0.7) in order to retain chemical groups.

Beside *in silico* models, also *in vitro* approaches are reported for the prediction of hepatotoxicity. Some of them are reviewed in Chen et al. (2014a,b). These *in vitro* assay-based models use human derived hepatocellular carcinoma cells (HepG2) to assess hepatotoxicity of drug/chemicals. The performance of these models in terms of sensitivity and specificity ranges from 45 to 100% and 82 to 100%, respectively. However, *in vitro* assays are time and money-consuming compared to *in silico* models.

## Conclusion

DILI is one of the main challenges for the pharmaceutical industry. Identifying easily substances that can interfere with the normal activity of the liver is essential in order to protect human health and reduce the money and efforts that drug development normally requires. Besides *in vitro* methods, increasing attention is now paid to computational methods used for the prediction of toxicity of substances. We present a SAR model for the prediction of hepatotoxicity induced by drugs, using experimental human data. We modeled the data in order to identify SAs for both hepatotoxicity and non-hepatotoxicity using both an automatic (SARpy software) and expert-based (based on chemical grouping) approach. The main effort was to model the negative property (non-hepatotoxicity). This is a new aspect of classification modeling, since most models are able to predict the toxicity while the safety of compounds is predicted only on the basis of the lack of information about the toxicity or is not-predicted at all. Future improvements could take place whenever more reliable data, mostly for non-hepatotoxicity (based on experimental results and not on the lack of activity), will be available. Considering the bias of the starting experimental data and the complexity of the endpoint, which comprises several mechanisms of action, the model we present here gave satisfactory results, higher than those already available. We built this model using human data so it could and be applied without any need for extrapolation from other species. Moreover, this model was tested on an external validation set, in order to evaluate its real predictive ability. This model will be freely available through the VEGA platform. It may help in the early identification and screening of drug candidates and in reduction of animals used for scientific purposes.

## Author contributions

FP: co-operation in development of research area and methodology; FP, AL, and EB: coordination in the manuscript preparation; AM: co-operation in chemical clustering analysis. All authors critically revised the manuscript and approved the final version.

### Conflict of interest statement

The authors declare that the research was conducted in the absence of any commercial or financial relationships that could be construed as a potential conflict of interest.

## Footnotes

^1^PubChem database, last access: 1 February 2016, https://pubchem.ncbi.nlm.nih.gov/.

^2^ChemAxon, Version 5.11.5, 2013, http://www.chemaxon.com.
