# Supplementary material for: A New Structure-Activity Relationship (SAR) Model for Predicting Drug-Induced Liver Injury, Based on Statistical and Expert-Based Structural Alerts
Source: Front Pharmacol. 2016 Nov 22;7:442. doi: 10.3389/fphar.2016.00442 (PMC5118449; doi:10.3389/fphar.2016.00442)
Supplement: Supplementary file 1 [file Table1.PDF]

Table 1.

The table below lists the 13 structural alerts (SAs) manually extracted. For each SA the SMART string, activity, chemical structure, total number of occurrences in the test and external validation sets and the number and percentage of true positive (TP), false positive (FP), true negative (TN) and false negative (FN) are shown.

We used Marvin for drawing and displaying chemical structures and substructures, Marvin 5.11.5, 2013, ChemAxon (http://www.chemaxon.com).

ID	SMARTS	Activity	Chemical structures	Pharmacolo gical class	Total occurrences in the test set	N. of TP **(%TP)**	N. FP **(%FP)**	Total occurrences in the external validation set	N. of TP **(%TP)**	N. FP **(%FP)**
1	[n,c]1ccn[n,c]c1	Hepatotoxic	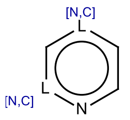	N-containing heterocycles aromatic compounds (pyridine, pyrazine, pyrimidine)	19	12 (63.16)	7 (36.84)	9	8 (88.89)	1 (11.11)
2	NS(=O)(=O)c1ccccc1	Hepatotoxic	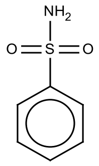	Sulphonamides	4	3 (75)	1 (25)	6	5 (83.33)	1 (16.67)
3	OC(=O)C1[C,S][S,O,C]C2CC(=O)N12	Hepatotoxic	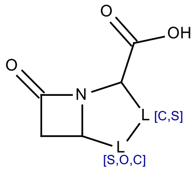	β-lactam antibiotics (penicillin)	1	1 (100)	0 (0)	0	0	0

4	O=C1N~CC=C[N,C]1C2C~[S,C]CO2	Hepatotoxic	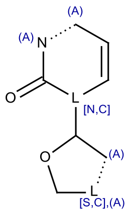	Nucleoside analogs	2	1<br>(50)	1<br>(50)	3	3<br>(100)	0<br>(0)
5	C1[S,C,N,O]c2ccccc2[N,C,S,O]c3ccccc13	Hepatotoxic	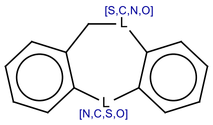	Tricyclic antidepressants (TCAs)	1	0<br>(0)	1<br>(100)	0	0	0
6	[N;!$([N+]);!$(NC=O);!$(N=[N,C,O])][a]	Hepatotoxic	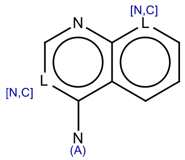	Aromatic amines	1	1<br>(100)	0<br>(0)	1	1<br>(100)	0<br>(0)
7	O=C1CCCCCCCCCCCCO1	Hepatotoxic	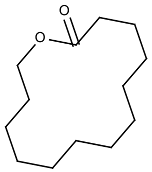	Macrolide antibiotics	3	3<br>(100)	0<br>(0)	0	0	0
8	Nc1[n,c]cc2C(=O)C(=CNc2[c,n]1)C(O)=O	Hepatotoxic	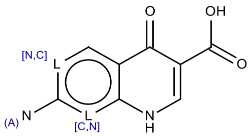	Anti-bacterial agents (fluorquinolone)	1	1<br>(100)	0<br>(0)	2	2<br>(100)	0<br>(0)

9	*N(*)CCC(c1cccc[n,c]1)c2cccc[n,c]2	Hepatotoxic	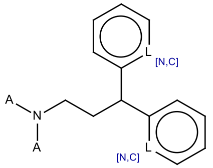	Cationic amphiphilic drugs (CADs)	0	0	0	0	0	0
10	CC=C(C)C=CC=C(C)C=C[R,a]	Hepatotoxic	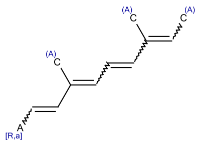	Retinoids	2	2 (100)	0 (0)	0	0	0
11	CNC(=O)N(CCCl)N=O	Hepatotoxic	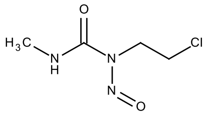	Nitrosourea compounds	0	0	0	0	0	0
12	C1CC2CCC3C(CC[C,c]4[C,c][C,c][C,c][C,c][C,c]34)C2C1	non-hepatotoxic	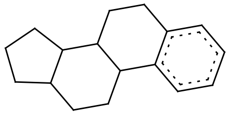	Steroids	3	1 (TN) (33.33)	2 (FN) (66.67)	1	1 (TN) (100)	0 (FN) (0)
13	CC(=O)NC1C2[S,O]CC=C(N2C1=O)C(O)=O	non-hepatotoxic	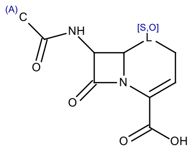	β-lactam antibiotics (cephalosporins)	4	3 (TN) (75.00)	1 (FN) (25.00)	0	0	0
