# Supplementary material for: A New Structure-Activity Relationship (SAR) Model for Predicting Drug-Induced Liver Injury, Based on Statistical and Expert-Based Structural Alerts
Source: Front Pharmacol. 2016 Nov 22;7:442. doi: 10.3389/fphar.2016.00442 (PMC5118449; doi:10.3389/fphar.2016.00442)
Supplement: Supplementary file 2 [file Table2.PDF]

Table 2.

The table below lists the 75 structural alerts (SAs) automatically extracted with SARpy software. For each SA the SMART string, activity, chemical structure, total number of occurrences in the training, test and external validation set and the number and percentage of true positive (TP), false positive (FP), true negative (TN) and false negative (FN) are shown.

We used Marvin for drawing and displaying chemical structures and substructures, Marvin 5.11.5, 2013, ChemAxon (http://www.chemaxon.com).

ID	SMARTS	Activity	Chemical structures	Total occurrences (training set)	N. of TP **(%TP)**	N. FP **(%FP)**	Total occurrences (test set)	N. of TP **(%TP)**	N. FP **(%FP)**	Total occurrences (external validation set)	N. of TP **(%TP)**	N. FP **(%FP)**
1	C(=CC(C)C)C CCCCCC	hepatotoxic	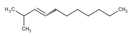	11	11 (100)	0 (0)	3	0 (0)	3 (100)	3	1 (33.33)	2 (66.67)
2	O=CC(NC)CO	hepatotoxic	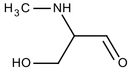	11	11 (100)	0 (0)	1	1 (100)	0 (0)	2	1 (50.00)	1 (50.00)
3	O(CCCC)CC(NC)CC	hepatotoxic	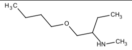	10	10 (100)	0 (0)	1	0 (0)	1 (100)	1	0 (0)	1 (100)
4	O=C(NCCCC) c1ccccc1	hepatotoxic	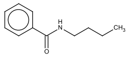	9	8 (88.88)	1 (11.12)	0	0	0	0	0	0
5	OCC(Oc1cccc c1)(C)C	hepatotoxic	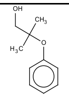	6	6 (100)	0 (0)	0	0	0	0	0	0
6	c1ccc(cc1)SC C	hepatotoxic	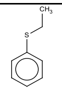	6	6 (100)	0 (0)	2	0 (0)	2 (100)	0	0	0
7	c1ccc(cc1)CC Cc2ccccc2	hepatotoxic	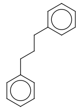	7	7 (100)	0 (0)	2	2 (100)	0 (0)	0	0	0
8	O=COC(CC(O)C)C(C)C	hepatotoxic	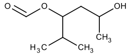	6	6 (100)	0 (0)	0	0	0	2	2 (100)	0 (0)
9	O=CCc1ccccc 1(N)	hepatotoxic	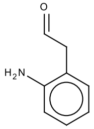	4	4 (100)	0 (0)	2	2 (100)	0 (0)	3	3 (100)	0 (0)
10	N(CC)CCCCN CC	hepatotoxic	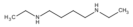	7	7 (100)	0 (0)	2	1 (50.00)	1 (50.00)	3	2 (66.67)	1 (33.33)
11	O=C(CO)C(O) CCO	hepatotoxic	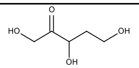	4	4 (100)	0 (0)	0	0	0	0	0	0
12	n2cnc1c(ncn1 CCOC)c2N	hepatotoxic	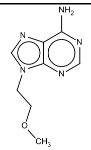	3	3 (100)	0 (0)	0	0	0	0	0	0
13	O(c1cccc(c1)C NCCCC)C	hepatotoxic	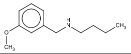	4	4 (100)	0 (0)	1	0 (0)	1 (100)	0	0	0
14	O=C(OCCc1c cccc1)C	hepatotoxic	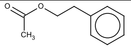	4	4 (100)	0 (0)	0	0	0	1	1 (100)	0 (0)
15	OCC(O)C(O)C CCNC	hepatotoxic	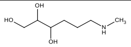	6	6 (100)	0 (0)	0	0	0	0	0	0

16	O=[N+]([O-])c1cccc(O)c1	hepatotoxic	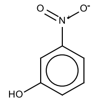	3	3 (100)	0 (0)	0	0	0	0	0	0
17	c1c(cc(cc1C)CC)C	hepatotoxic	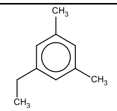	5	4 (80.00)	1 (20.00)	0	0	0	1	1 (100)	0 (0)
18	O=CNC(CC=O)CCC	hepatotoxic	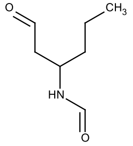	3	3 (100)	0 (0)	0	0	0	0	0	0
19	O=CNC(C(=O)O)CCC	hepatotoxic	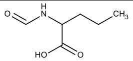	9	9 (100)	0 (0)	0	0	0	0	0	0
20	O=SCCNCCC	hepatotoxic	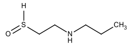	3	3 (100)	0 (0)	0	0	0	0	0	0
21	O=C(N)N(N)CC	hepatotoxic	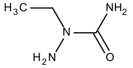	5	4 (80.00)	1 (20.00)	0	0	0	2	2 (100)	0 (0)
22	FC(F)CCl	hepatotoxic	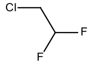	3	3 (100)	0 (0)	0	0	0	0	0	0
23	O=C(NC)CCc1ccccc1	hepatotoxic	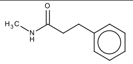	13	12 (92.30)	1 (7.70)	2	1 (50.00)	1 (50.00)	1	0 (0)	1 (100)
24	O=COC(C)COC	hepatotoxic	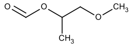	11	10 (90.90)	1 (9.10)	4	2 (50.00)	2 (50.00)	1	1 (100)	0 (0)
25	Oc1ccc(c(OC)c1)C	hepatotoxic	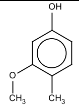	12	9 (75.00)	3 (25.00)	4	3 (75.00)	1 (25.00)	1	1 (100)	0 (0)
26	N(C)C(C)CNCCCC	hepatotoxic	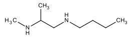	20	18 (90.00)	2 (10.00)	5	4 (80.00)	1 (20.00)	4	2 (50.00)	2 (50.00)
27	NCCCCCNC	hepatotoxic	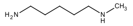	26	22 (84.61)	4 (15.39)	5	2 (40.00)	3 (60.00)	4	2 (50.00)	2 (50.00)
28	O=CC(N)Cc1ccccc1	hepatotoxic	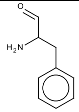	17	14 (82.35)	3 (17.65)	3	2 (66.67)	1 (33.33)	2	1 (50.00)	1 (50.00)
29	c1ccc(c(c1)C=CC)C	hepatotoxic	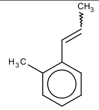	13	10 (76.92)	3 (23.08)	3	3 (100)	0 (0)	2	2 (100)	0 (0)
30	n1cc[nH]c1	hepatotoxic	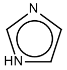	13	11 (84.61)	2 (15.39)	2	0 (0)	2 (100)	2	1 (50.00)	1 (50.00)

31	NCNc1ccccc1	hepatotoxic	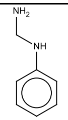	12	9 (75.00)	3 (25.00)	3	3 (100)	0 (0)	2	1 (50.00)	1 (50.00)
32	O=CC(c1ccccc1)CN	hepatotoxic	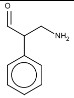	6	5 (83.33)	1 (16.67)	0	0	0	0	0	0
33	FC(F)(F)c1ccccc1	hepatotoxic	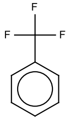	7	6 (85.71)	1 (14.29)	4	2 (50.00)	2 (50.00)	1	1 (100)	0 (0)
34	n1ccccc1Cc2ccccc2	hepatotoxic	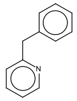	4	3 (75.00)	1 (25.00)	0	0	0	1	0 (0)	1 (100)
35	N(CC)CCCl	hepatotoxic	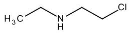	6	5 (83.33)	1 (16.67)	3	2 (66.67)	1 (33.33)	0	0	0
36	c1cc(C)sc1	hepatotoxic	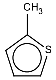	14	10 (71.42)	4 (28.58)	3	3 (100)	0 (0)	2	2 (100)	0 (0)
37	C(=C(Cl))	hepatotoxic	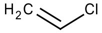	4	3 (75.00)	1 (25.00)	3	1 (33.33)	2 (66.67)	0	0	0
38	O=S(=O)(N)c1ccccc1	hepatotoxic	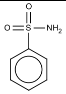	31	22 (70.96)	9 (29.04)	4	3 (75.00)	1 (25.00)	6	5 (83.33)	1 (16.67)
39	Nc1ccc(cc1)S(=O)=O	hepatotoxic	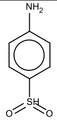	16	12 (75.00)	4 (25.00)	3	2 (66.67)	1 (33.33)	2	2 (100)	0 (0)
40	[n,o]1n[c,n][c,n,s,nH][c,n]1	hepatotoxic	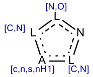	34	24 (70.58)	10 (29.42)	8	3 (37.50)	5 (62.50)	5	5 (100)	0 (0)
41	OC(c1ccccc1)c2ccccc2	non-hepatotoxic	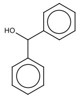	6	6 (TN)	0 (FN) (0)	1	1 (TN) (100)	0 (FN) (0)	2	2 (TN) (100)	0 (FN) (0)
42	O=C(O)CCc1ccc(OC)cc1	non-hepatotoxic	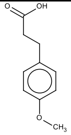	7	6 (TN) (85.71)	1 (FN) (14.29)	0	0	0	0	0	0

43	OC1OC(CN)CCC1(N)	non-hepatotoxic	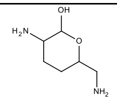	6	6 (TN) (100)	0 (FN) (0)	0	0	0	0	0	0
44	OCCNC(C)(C)C	non-hepatotoxic	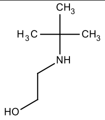	6	6 (TN) (100)	0 (FN) (0)	1	1 (TN) (100)	0 (FN) (0)	0	0	0
45	O=C(OC2CCC3C4CCc1cc(O)ccc1C4(CCC23(C)))CC	non-hepatotoxic	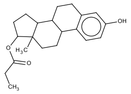	5	5 (TN) (100)	0 (FN) (0)	0	0	0	0	0	0
46	O=C(N(c1ccccc1)CC)C	non-hepatotoxic	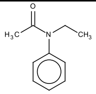	7	7 (TN) (100)	0 (0)	2	2 (TN) (100)	0 (FN) (0)	1	0 (TN) (0)	1 (FN) (100)
47	O=C(Nc1ccccc1C)CC	non-hepatotoxic	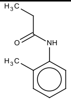	4	4 (TN) (100)	0 (FN) (0)	2	2 (TN) (100)	0 (FN) (0)	0	0	0
48	O=COCC(C)(C)COC=O	non-hepatotoxic	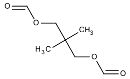	4	4 (TN) (100)	0 (FN) (0)	0	0	0	0	0	0
49	Oc4ccc1c2c4(OC3CCCC(O)(C(N(C)CC)C1)C23(C))	non-hepatotoxic	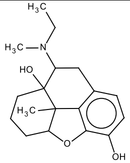	3	3 (TN) (100)	0 (FN) (0)	0	0	0	1	0 (TN) (0)	1 (FN) (100)
50	O=C(c1ccccc1)c2ccccc2(O)	non-hepatotoxic	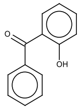	3	3 (TN) (100)	0 (FN) (0)	0	0	0	1	0 (TN) (0)	1 (FN) (100)
51	c1ccc(cc1)Cc2ccccc2Cl	non-hepatotoxic	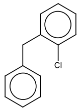	4	4 (TN) (100)	0 (FN) (0)	1	0 (TN) (0)	1 (FN) (100)	0	0	0
52	O=C(NC)CNC(=O)C(CC)CCC	non-hepatotoxic	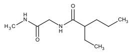	3	3 (TN) (100)	0 (FN) (0)	1	1 (TN) (100)	0 (FN) (0)	0	0	0
53	OC(c1cccc(OC)c1)CNC	non-hepatotoxic	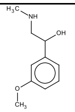	4	4 (TN) (100)	0 (FN) (0)	0	0	0	0	0	0
54	O(c1ccccc1)CCN(C)CC	non-hepatotoxic	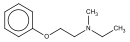	3	3 (TN) (100)	0 (FN) (0)	0	0	0	3	0 (TN) (0)	3 (FN) (100)

55	Oc1ccc(cc1)NCCCC	non-hepatotoxic	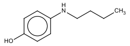	3	3 (TN) (100)	0 (FN) (0)	0	0	0	0	0	0
56	OCCC1(C)(CCCCC1)	non-hepatotoxic	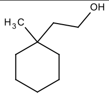	3	3 (TN) (100)	0 (FN) (0)	4	3 (75.00)	1 (25.00)	1	0 (TN) (0)	1 (FN) (100)
57	c1ccc(c(c1)CCC)Cl	non-hepatotoxic	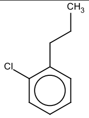	4	4 (TN) (100)	0 (FN) (0)	1	1 (TN) (100)	0 (FN) (0)	0	0	0
58	C1C2CC3CC1CC(C2)C3	non-hepatotoxic	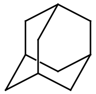	3	3 (TN) (100)	0 (FN) (0)	0	0	0	0	0	0
59	N1CN(C)CC(C)C1	non-hepatotoxic	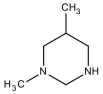	3	3 (TN) (100)	0 (FN) (0)	0	0	0	0	0	0
60	OCCSCCC	non-hepatotoxic	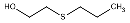	3	3 (TN) (100)	0 (FN) (0)	0	0	0	0	0	0
61	O=S(=O)(NC)C	non-hepatotoxic	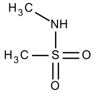	3	3 (TN) (100)	0 (FN) (0)	0	0	0	0	0	0
62	O=Cc1cccc(c1)NCC	non-hepatotoxic	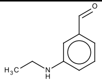	8	8 (TN) (100)	0 (FN) (0)	1	1 (TN) (100)	0 (FN) (0)	0	0	0
63	OCCC(CO)CCO	non-hepatotoxic	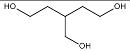	6	6 (TN) (100)	0 (FN) (0)	0	0	0	1	0 (TN) (0)	1 (FN) (100)
64	O=C(N(c1ccccc1C)C)C	non-hepatotoxic	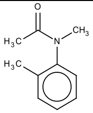	8	7 (TN) (87.50)	1 (FN) (22.50)	1	1 (TN) (100)	0 (FN) (0)	1	0 (TN) (0)	1 (FN) (100)
65	O=CC(C)(C)CN	non-hepatotoxic	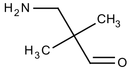	12	10 (TN) (83.33)	2 (FN) (16.67)	0	0	0	0	0	0
66	CC[N+](C)(C)C	non-hepatotoxic	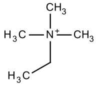	12	10 (TN) (83.33)	2 (FN) (16.67)	4	4 (TN) (100)	0 (FN) (0)	4	4 (TN) (100)	0 (FN) (0)
67	O=C1CCCCC1	non-hepatotoxic	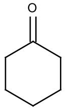	10	8 (TN) (80.00)	2 (FN) (20.00)	2	0 (TN) (0)	2 (FN) (100)	1	0 (TN) (0)	1 (FN) (100)

68	c1ccc(cc1)I	non-hepatotoxic	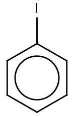	15	13 (TN) (86.66)	2 (FN) (13.34)	1	1 (TN) (100)	0 (FN) (0)	1	0 (TN) (0)	1 (FN) (100)
69	O=C(N)c1cccc (c1)S(=O)=O	non-hepatotoxic	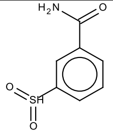	4	3 (TN) (75.00)	1 (FN) (25.00)	0	0	0	0	0	0
70	Nc1ccc2ccccc 2(c1)	non-hepatotoxic	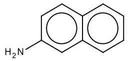	4	3 (TN) (75.00)	1 (FN) (25.00)	0	0	0	0	0	0
71	O(c1ccc(cc1)C)CCC	non-hepatotoxic	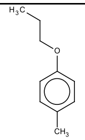	10	7 (TN) (70.00)	3 (FN) (30.00)	2	0 (TN) (0)	2 (FN) (100)	0	0	0
72	OC1OCC(O)C (O)C1(N)	non-hepatotoxic	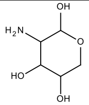	9	7 (TN) (77.77)	2 (FN) (22.23)	2	1 (TN) (50.00)	1 (FN) (50.00)	2	1 (TN) (50)	1 (FN) (50)
73	c1cc(c(cc1Cl) Cl)C	non-hepatotoxic	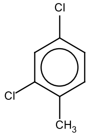	9	7 (TN) (77.77)	2 (FN) (22.23)	1	0 (TN) (0)	1 (FN) (100)	0	0	0
74	NN=C	non-hepatotoxic	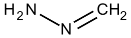	10	7 (TN) (70.00)	3 (FN) (30.00)	2	1 (TN) (50.00)	1 (FN) (50.00)	4	0 (TN) (0)	4 (FN) (100)
75	CC(=O)Nc1cc ccc1C	non-hepatotoxic	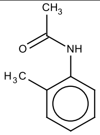	18	14 (TN) (77.77)	4 (FN) (22.23)	2	2 (TN) (100)	0 (FN) (0)	1	0 (TN) (0)	1 (FN) (100)
